# Research on Error Point Deletion Technique in Three-Dimensional Reconstruction of ISAR Sequence Images

**DOI:** 10.3390/s25061689

**Published:** 2025-03-08

**Authors:** Mingyu Ma, Yingni Hou

**Affiliations:** 1 Nanjing Research Institute of Electronics Technology, Nanjing 210039, China; houyingni@163.com; 2 National Key Laboratory of Radar Detection and Sensing, Nanjing 210039, China; 3 Jiangsu Provincial Key Laboratory of Detection and Sensing Technology, Nanjing 210039, China

**Keywords:** inverse synthetic aperture radar (ISAR), feature point matching, error point deletion, motion vector, point cloud density, three-dimensional (3D) imaging

## Abstract

Three-dimensional reconstruction using a two-dimensional inverse synthetic aperture radar (ISAR) faces dual challenges: geometric distortion in initial point clouds caused by accumulated feature-matching errors and degraded reconstruction accuracy due to point cloud outlier interference. This paper proposes an optimized method to delete the error points based on motion vector features and local spatial point cloud density. Before reconstruction, feature point extraction and matching for ISAR sequence images are performed using Harris corner detection and the improved Kanade–Lucas–Tomasi (KLT) algorithm. To address the issue of mismatched points, a method based on motion vector features is proposed. This method applies the dual constraints of motion distance and direction thresholds and deletes mismatched points based on local motion consistency. After point cloud reconstruction, a clustering method based on local spatial point cloud density is employed to effectively remove outliers. To validate the effectiveness of the proposed method, simulation experiments comparing the performance of different approaches are conducted. The experimental results demonstrate the effectiveness and robustness of the proposed method in the 3D reconstruction of moving targets.

## 1. Introduction

Radar imaging technology primarily encompasses two typical systems: the synthetic aperture radar (SAR) and inverse synthetic aperture radar (ISAR). The imaging principles of the ISAR and SAR are fundamentally the same. In the range dimension, both achieve high resolution by employing broadband radar signals. In the azimuth dimension, both utilize the relative motion between the radar and the target to form a synthetic aperture for high-resolution imaging. However, there are some differences between the SAR and ISAR. SAR imaging involves radar motion to image stationary scenes, whereas ISAR imaging involves the radar stationary observation of moving targets. Additionally, the ISAR requires the estimation and compensation of the target’s translational components from the radar echoes, retaining only the rotational components for imaging. In contrast, the SAR directly constructs the synthetic aperture through the precise control of the radar platform’s motion.

The inverse synthetic aperture radar (ISAR) generates two-dimensional high-resolution images by transmitting wideband signals and using pulse compression techniques. Two-dimensional imaging is widely used in military and civilian applications. However, 2D ISAR looks like a projection of the target on a 2D plane, which lacks physical information such as the shape and size of the target. Therefore, it is necessary to use 3D imaging techniques to obtain physical information such as a more stable shape and structure of the target. There are two main directions of research on the ISAR target 3D reconstruction problem. The first is an interferometric inverse synthetic aperture radar combining ISAR imaging and interferometric processing techniques for imaging [1,2,3,4,5]. The interferometric ISAR system is designed with two antennas, and the two antennas surround a plane to form an interferometric ISAR system to realize the three-dimensional ISAR imaging of the target [5]. However, this imaging method requires multiple radars to observe, which has higher system requirements and is more difficult to realize. The second method is based on the existing single-station radar, which can obtain the two-dimensional ISAR image sequence of the target by observing the target for a long time and then process the sequence images and realize the 3D reconstruction of the target through the algorithm. Therefore, how to obtain more information about the physical characteristics of the target by using a series of 2D ISAR images for the 3D reconstruction of the target has important research value in military and other fields.

Before using sequence images for 3D reconstruction, a key step is to extract and match features for each frame in the image sequence. In [6], the 3D reconstruction of a target is performed by extracting the geometric features of the target and utilizing the contours of the target. The commonly used feature point extraction algorithms are the Harris corner point detection algorithm [7], Scale-Invariant Feature Transform (SIFT) and its improved methods [8,9], the Speeded-Up Robust Features (SURF) algorithm [10,11], etc. Di [12] used the scattering center association matching method based on the SURF algorithm to extract and correlate features of the sequence images. Yang [13] used the SIFT algorithm to extract the feature point information of scattering points and used the Randomized Sampling Consistency Algorithm (RANSAC) method to match feature points of the ISAR sequence images. However, a small number of matching points affect the 3D reconstruction effect of the ISAR images, which is also a common problem faced by point matching technology between images. The SIFT algorithm was improved in [14] to improve the matching efficiency by constructing feature descriptors from distance vectors, angles, and signal-to-noise ratios between scatterers. Xu [15] proposed an image matching method based on extracted dominant scatterers, which used scattering center extraction to convert radar images into scattering point sets. The scatterer was then coarse-matched using the RANSAC method and fine-matched by iteratively estimating the scatterer heights and exact affine transformation parameters. Guo [16] improved the RANSAC and deleted the error points by adding quality constraints, that is, the ratio of maximum angle to minimum angle was used to measure whether the error points were internal points. Wang [17] directly used the KLT feature tracking algorithm in the extraction of 2D image feature points to realize the correlation between adjacent images. In [18], the KLT algorithm was improved to achieve more accurate correlation between images. Zhou [19] used ISAR image sequence energy accumulation (ISEA) for three-dimensional geometric reconstruction. In recent years, some researchers have introduced some methods of deep learning for feature extraction and matching [20,21].

In the aspect of 3D reconstruction, the 3D reconstruction of a spatial target can be realized by using the relationship between the 3D spatial target and 2D image imaging. The orthogonal decomposition method can reconstruct the geometric shape of the initial target by using the orthogonal decomposition relationship between the two, so the factorization method can be used for three-dimensional reconstruction [22]. However, because the matrix factorization factor is not unique, there will be errors in the reconstructed 3D position coordinates and other information. Zhou [21] proposed an extended factorization framework based on motion theory, combined with 3D reconstructed coordinates and instantaneous projection vectors for modeling, and obtained 3D point clouds by the quantum-behaved particle swarm optimization algorithm. Xu [23] constrained the projection vector to obtain the instantaneous pose of the target and combined the scattering point information from different perspectives to carry out 3D reconstruction. In [24], the mapping between the morphological structure of the space target and the scattering of ISAR imaging was established, and the 3D structure information of the space target was obtained by reconstruction based on the projection morphology of the sequence images. Zhou proposed a new algorithm for iteratively scaling the lateral distance coordinates by combining the lateral projection vector and the distance projection vector, which can improve the accuracy of target 3D reconstruction [25]. Wang used the projection equation to back-project the feature points into 3D space and applied the Iterative Closest Point algorithm to fuse multiple 3D point clouds, thereby restoring the 3D structure of the target [26].

**Figure 1:**
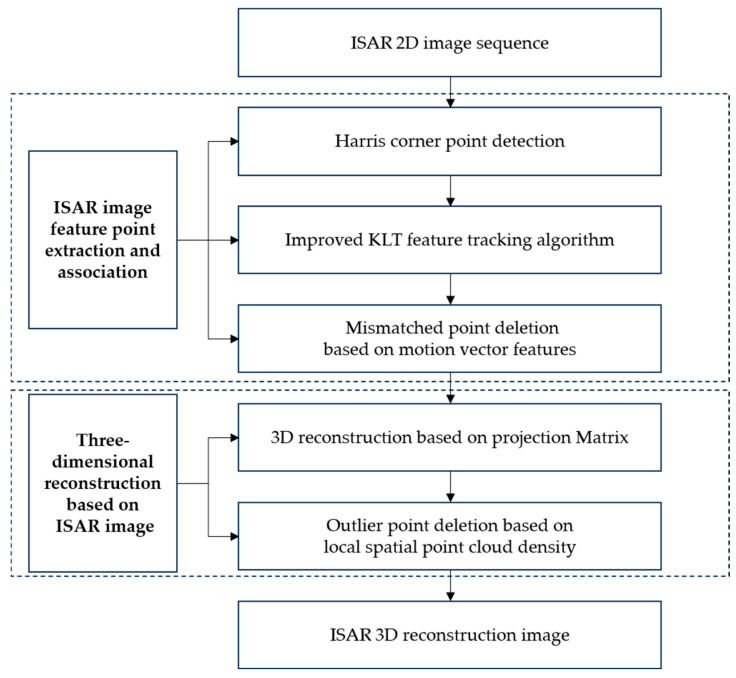
Block diagram of the research content.

This paper mainly studies the deletion of the error point in the process of 3D reconstruction based on ISAR sequence images and proposes a method to delete the error point after matching the sequence images and 3D reconstruction, respectively. Firstly, feature points are extracted by the Harris corner detection algorithm, and the feature points in subsequent frames are tracked and matched by the improved KLT algorithm. For the problem of the deletion of mismatched points, a method of deleting mismatched points based on the motion feature vector is proposed. The method mainly uses the motion distance and direction of the matched point pairs of two neighboring frames in the local area to delete the mismatched points. After that, the sequence images are reconstructed in 3D. For the problem of deleting the remaining error points after reconstruction, this paper proposes a method to delete a small number of error points in the space based on the density of the local point cloud and verifies the feasibility of the method through simulation experiments. The block diagram of the research content in this paper is shown in Figure 1.

The subsequent sections are structured as follows. Section 2 introduces the principle of ISAR 3D imaging based on the projection matrix. Section 3 presents 3D reconstruction based on ISAR sequence images. Section 4 describes, in detail, the proposed method for error point deletion. Section 5 conducts simulation experiments of satellite models. Finally, Section 6 gives the conclusion.

## 2. Principles of ISAR 3D Imaging Based on Projection Matrix

**Figure 2:**
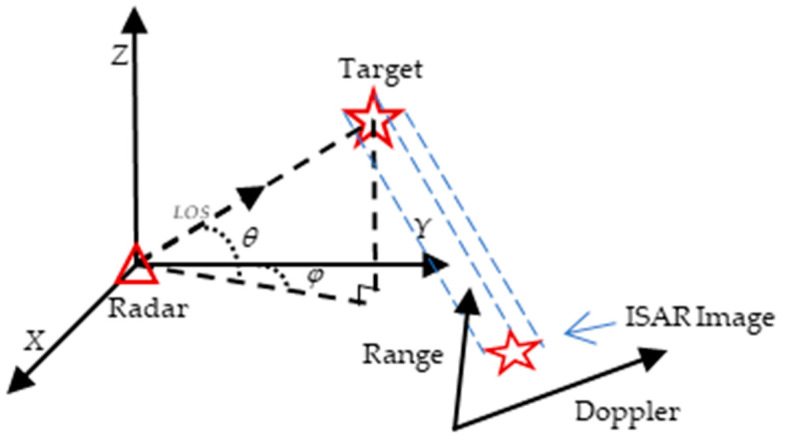
Imaging observation geometry.

The imaging plane consists of distance and azimuth vectors through orthogonalization, and the corresponding projection matrix consists of distance and azimuth vectors. In order to solve the projection matrix, the geometric projection model for ISAR imaging is first established. Figure 2 shows the projection process of an airborne target, where θ and φ are the pitch angle and azimuth angle of the radar line of sight, respectively, the triangle represents the radar, and the pentagram is the target.

Radar LOS direction unit vector $\vec{k}$ and target unit vector $\vec{l}$ are, respectively,

(1) $$\vec{k}=\left[\begin{array}{c} \cos\theta \sin\varphi \\ \cos\theta \cos\varphi \\ \sin\theta \end{array}\right]$$

(2) $$\vec{l}=\left[\begin{array}{c} \cos\alpha \sin\beta \\ \cos\alpha \cos\beta \\ \sin\alpha \end{array}\right]$$

The range dimension resolution and Doppler dimension resolution of the ISAR image are, respectively,

(3) $$\Delta r=\frac{c}{2B}$$

(4) $$\Delta f_{a}=\frac{\lambda }{2\Delta \theta }$$

where B is the bandwidth of the radar-transmitted signal, c is the speed of light, Δθ is the angle of the target relative to the radar line of sight, and λ is the signal wavelength. The expression of the radar projection imaging process is as follows:

(5) $$\left(\begin{array}{c} r\\ d \end{array}\right)=\vec{A}\cdot \vec{l}=\left(\begin{array}{c} \vec{m}\\ \vec{n} \end{array}\right)\cdot \left(\begin{array}{c} x\\ y\\ z \end{array}\right)$$

(6) $$\vec{A}=\left[\begin{array}{c} \vec{m}\\ \vec{n} \end{array}\right]=\left[\begin{array}{c} \begin{array}{ccc} \frac{\cos\theta \sin\varphi }{\Delta r} & \frac{\cos\theta \cos\varphi }{\Delta r} & \frac{\sin\theta }{\Delta r} \end{array}\\ \begin{array}{ccc} -\frac{-\sin\theta \sin\varphi \theta^{\prime }+\cos\theta \cos\varphi \varphi^{\prime }}{\Delta f_{a}} & -\frac{\sin\theta \cos\varphi \theta^{\prime }+\cos\theta \sin\varphi \varphi^{\prime }}{\Delta f_{a}} & -\frac{\cos\theta \theta^{\prime }}{\Delta f_{a}} \end{array} \end{array}\right]$$

where ${\left(r,d\right)}^{T}$ is the coordinate of the point within the RD image, $\vec{A}$ is the projection matrix, the direction vectors $\vec{m}$ and $\vec{n}$ represent the range and Doppler dimension directions of the imaging plane, respectively, $\theta^{\prime }$ is the instantaneous pitch angle speed, and $\varphi^{\prime }$ is the instantaneous azimuth angle speed.

In the process of observation, the projection matrix $\vec{A}$ can be obtained from $\vec{m}$ and $\vec{n}$. Through the inverse matrix operation, the three-dimensional coordinates of the points can be obtained, that is,

(7) $$\left(\begin{array}{c} x\\ y\\ z \end{array}\right)={\vec{A}}^{-1}\cdot \left(\begin{array}{c} r\\ d \end{array}\right)$$

According to the above method, ISAR 3D imaging can be realized by using the projection matrix to invert the coordinates of the target in the 2D ISAR image in 3D space.

## 3. Three-Dimensional Reconstruction Based on ISAR Images

### 3.1. ISAR Image Feature Point Extraction

Before reconstruction, the feature points in the image need to be extracted and associated. For the extraction of feature points, this paper uses the Harris corner detection algorithm.

Harris corner detection identifies corners based on significant changes in grayscale, and in ISAR images, target scattering points also exhibit noticeable variations. Therefore, Harris can effectively extract strong scattering points in ISAR images. Furthermore, the Harris algorithm has low computational complexity and a short processing time. Other feature extraction algorithms, such as SIFT and SURF, can yield more stable feature points; however, they are computationally complex. When using SIFT and SURF for subsequent image feature matching, the number of successfully matched points tends to be lower, which can reduce the accuracy of 3D reconstruction results. Therefore, the Harris corner detection algorithm is used to extract the feature points in the image.

The Harris corner point detection algorithm determines the location of the corner point by calculating the corner point response function of each pixel in the image, and in addition, by comparing the response function of its corner point with that of the surrounding pixels, the local maximum is retained as the corner point for non-maximal value suppression [27]. In this paper, the Harris corner point detection algorithm is used to extract the point features in the first frame of the image, and it can extract the target feature points in the image.

### 3.2. ISAR Image Feature Point Association

For the association of feature points between subsequent ISAR images, this paper uses the improved KLT algorithm to track the feature points in the image of the next frame, so as to realize the association of feature points between two neighboring frames. The forward and backward matching method is used for preliminary false match point deletion to obtain the set of matching points between multi-frame images.

The KLT (Kanade–Lucas–Tomasi) matching algorithm was proposed by Lucas and Kanade et al. and improved by subsequent researchers. The algorithm first extracts the feature points in the first frame of the image, then tracks these feature points in the second frame of the image, and finally realizes the matching of feature points in the two frames. The core principle is to find the best matching feature points by calculating the sum of squares of the gray level differences in the images. The algorithm is based on three basic assumptions: the brightness of the image remains constant between two neighboring frames; the motion changes in the image are small; and the neighboring pixel points have similar motion trends.

The main steps of the improved KLT algorithm are as follows:

1. Pyramid building

The images I and J are decomposed into K-layer pyramids and each layer of the image is obtained by downsampling. Let $I_{k}$ and $J_{k}$ denote the representation of images I and J on the kth layer pyramid, respectively, where k=0,1,…,K, and $I_{0}$, $J_{0}$ are the original images.

2. Optical flow estimation

In the uppermost layer k=K, the optical flow is calculated. The integral expression for the gray scale change is given by

(8) $$\varepsilon (d)=\iint {\left[J(P+d)-I(P)\right]}^{2}w(P)dP$$

It is assumed that the gray values of the two adjacent frames of image pixels are constant, but this may not be true in reality, so the minimum value of gray change needs to be solved. Therefore, there is

(9) $$\frac{\partial \varepsilon (d)}{\partial d}=\iint J(P)-I(P)+g^{T}d]gw(P)dP=0$$

where $g={\left[\frac{\partial J(P)}{\partial x},\frac{\partial J(P)}{\partial y}\right]}^{T}$.

The final simplification yields

(10) Zd=e

where $Z=\iint gg^{T}w(P)dP$, e=∬[J(P)−I(P)]⋅g⋅w(P)dP.

The d value is obtained by the above formula.

3. Affine estimation

In neighboring frame images, the target may be geometrically distorted in 2D ISAR images, and Jianbo Shi proposed to introduce the affine matrix for tracking optical images [28]. In this paper, affine transformation is introduced to improve the accuracy of tracking. Assuming that the coordinates of a point in the image are (x,y), and the coordinates of the corresponding point in the next frame of the image are $\left(x^{\prime },y^{\prime }\right)$, the equation of the affine transformation between the two is

(11) $$\left(\begin{array}{c} x^{\prime }\\ y^{\prime } \end{array}\right)=D\cdot \left(\begin{array}{c} x\\ y \end{array}\right)+d=\left(\begin{array}{cc} d_{xx} & d_{xy}\\ d_{yx} & d_{yy} \end{array}\right)\cdot \left(\begin{array}{c} x\\ y \end{array}\right)+\left(\begin{array}{c} d_{x}\\ d_{y} \end{array}\right)$$

where D is the deformation matrix and d is the translation.

4. Dynamic window adjustment

The size of the local window has a significant impact on tracking performance. An overly large window may result in increased computational load and susceptibility to noise interference, while a window that is too small may lack sufficient information to accurately track the features. To address this issue, a dynamic window adjustment method is employed to adaptively regulate the window size. The dynamic window adjustment primarily adjusts the size of the local window based on the proportion of effective feature points in the current image, that is, by adjusting the local window size according to the ratio of successfully tracked feature points to the total number of feature points.

5. Pyramid-layered iterations

Starting from the Kth layer, the optical flow is computed layer by layer downwards. For the kth layer (k=K−1,K−2,…,0), the optical flow of the current layer is calculated using the optical flow computed on the previous layer as the initial value. That is, the result on the high-resolution layer is used as the initial value on the low-resolution layer.

6. Forward and backward matching

The feature coarse matching phase uses a forward–backward matching strategy based on the Euclidean distance to obtain initial matching pairs. Specifically, after obtaining the feature points in the subsequent frame, these feature points are tracked backward to the previous frame. For the feature point P1 in the image of the previous frame, the reverse tracking algorithm is used to find the corresponding feature point P2 in the current frame’s image. To determine whether these two feature points are the same feature point in the image, the Euclidean distance is used as a measure. If the distance between P1 and P2 is far, it is considered that these two feature points are not the same feature point and are deleted. By this method, we can initially delete the mismatched points.

## 4. Error Point Deletion

In order to improve the accuracy of 3D reconstruction, this paper deletes the error points. For inter-image matching before reconstruction, a method based on motion vector features is proposed to delete the mismatched points in the matched pairs, and the motion distance and direction between the matched pairs are used to filter out the error points. In addition, for the outlier points existing after reconstruction, the method of using local point cloud density is proposed to perform error point deletion, and the outlier points appearing in the local region are deleted.

### 4.1. Mismatched Point Deletion Based on Motion Vector Features

It is common to use the distance information between image features to match the feature points. However, by matching image features only by this feature, the matching condition is single, and it is easy for the matching error to occur. In order to solve the problem that there are still some associated error points in the point set after association, this paper proposes to use the motion vector features of the feature points to fine-match the matched point set and further determine whether any mismatched points occurred by the motion distance and direction of the points.

In the two frames of images, the motion of the feature points is similar. By analyzing the motion vector of the associated feature points, the mismatched feature points can be further deleted. The length of the line between the matching points can represent the distance of the feature points. The slope of the line can be manipulated to represent the direction in which the point is moving. In this paper, these two features are used for further mismatched points’ deletion.

**Figure 3:**
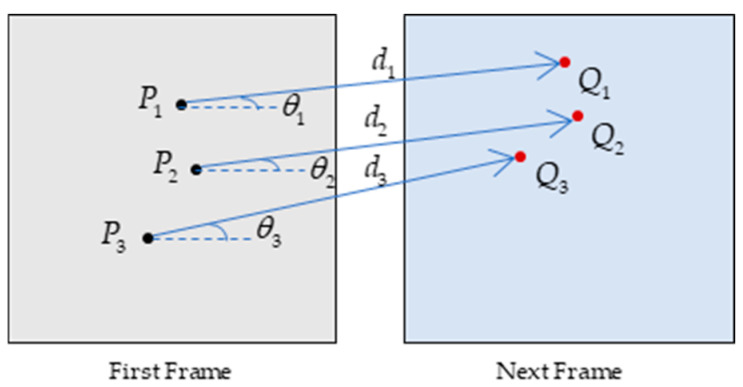
Motion vector diagram.

The schematic diagram of the algorithm is shown in Figure 3, where P is the point in the first frame, and Q is the corresponding point in the next frame. The motion vector V of the point between the two frames of the image can be represented by the distance d and the direction angle θ.

Specifically, it is assumed that P(m) is a feature-matching point in the images of two neighboring frames, where m=1,2,…,f, f is the total number of frames. Then, the formulae for the distance $V_{d_{i}}$ between the matching point pairs of the two neighboring frames and the slope $V_{k_{i}}$ of the connecting line are, respectively,

(12) $$V_{d_{i}}=\sqrt{{\left(P{\left(m+1\right)}_{x_{i}}-P{\left(m\right)}_{x_{i}}\right)}^{2}+{\left(P{\left(m+1\right)}_{y_{i}}-P{\left(m\right)}_{y_{i}}\right)}^{2}}$$

(13) $$V_{k_{i}}=\frac{P{\left(m+1\right)}_{y_{i}}-P{\left(m\right)}_{y_{i}}}{P{\left(m+1\right)}_{x_{i}}-P{\left(m\right)}_{x_{i}}}$$

where i=1,2,…,n is the corresponding matching feature point pair, n is the total number of matching point pairs, and $\left(x_{i},y_{i}\right)$ is the coordinate of the feature point.

If the slope is chosen to represent the direction of motion, there will be a problem of processing positive and negative values, so it converts it into an angle to represent the direction of point motion. The angle $V_{\theta_{i}}$ is

(14) $$V_{\theta_{i}}=atan2(P{\left(m+1\right)}_{y_{i}}-P{\left(m\right)}_{y_{i}},P{\left(m+1\right)}_{x_{i}}-P{\left(m\right)}_{x_{i}})$$

The process of deleting mismatched points based on motion vectors is as follows:

1. Initialize the set of matching points, where the set of connecting distances and the angles of the directions of matched feature point pairs are $V_{d}$ and $V_{\theta }$.
2. Set the size of the window to be used for filtering the mismatched points in the local region.
3. Set the adaptive threshold according to the mean value of non-zero pixels in the window and the factor; the specific formula is
  (15) $$T=\alpha \cdot \frac{1}{\sum_{j=1}^{s}1(pixel[j]\neq 0)}\sum_{i=1}^{r}pixel[N_{i}]$$
  where α is the scaling factor, pixel is the pixels within the window, $\sum_{i=1}^{r}pixel[N_{i}]$ is the sum of non-zero pixels, r is the number of non-zero pixels, and 1(pixel[j]≠0) is the indicator function, which is 1 for pixel[j]≠0 and 0 otherwise.
4. Determine $V_{d}$ and $V_{\theta }$ and their thresholds, respectively. When the matching point pair meets, and the numerical size is less than the threshold, retain its matching point pair, and otherwise, delete it.

The accuracy of the set of matching points is improved by the secondary optimization of matching points through the above process.

### 4.2. Outlier Point Deletion Based on Local Spatial Point Cloud Density

The method proposed in the previous section is to process the error points after image correlation matching before reconstruction, and some error points still exist after 3D reconstruction. In this paper, by analyzing the local spatial density of each point in the point cloud, the points with abnormal density are identified and deleted.

The main parameters are the search radius R and the density threshold T. The search radius determines the size of the range that needs to be considered when searching for neighboring points. The local density is calculated by the following equation:

(16) $$\rho_{i}=\sum_{j\in N_{i}}w_{j}\cdot f_{j}$$

where $N_{i}$ is the set of neighboring points within a radius centered at point $P_{i}$. The weight $w_{j}$ is based on a function of the distance $d_{i,j}$ between point $P_{i}$ and point $P_{j}$, and is often computed as a Gaussian function:

(17) $$w_{j}=\exp(-{\left(\frac{d_{i,j}}{\sigma }\right)}^{2})$$

where σ is a parameter controlling the rate of weight decay.

The process of deleting outlier points based on point cloud density is as follows:

1. Input: Point cloud data.
2. Initialization: Search radius R; density threshold T.
3. Calculation of local density: For each point in the point cloud, use the search radius R to search for neighboring points around it. Then, count the number of neighbor points searched and calculate the local density of the point.
4. Determination of outlier: Travel through all the points in the point cloud, compare their local densities with the set density threshold, and mark the points whose local density is lower than the threshold as outliers.
5. Deletion of error points: Delete all the points labeled as outliers from the point cloud.
6. Output: Deleted point cloud data.

## 5. Experiments and Results

In this section, simulation experiments are performed to verify the effectiveness of the proposed method. The experiments take the satellite as the target and perform 3D reconstruction of the 2D ISAR sequence images under the condition of a high signal-to-noise ratio (SNR), and the SNR of the image is 10 dB.

**Figure 4:**
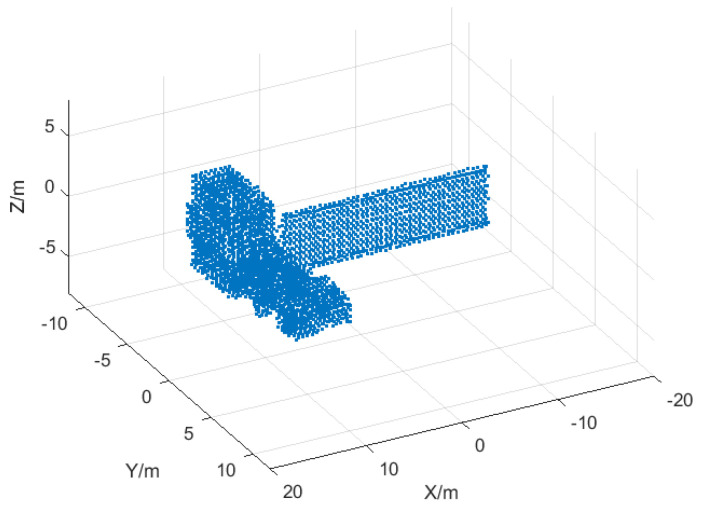
Three-Dimensional image of the satellite model.

**Figure 5:**
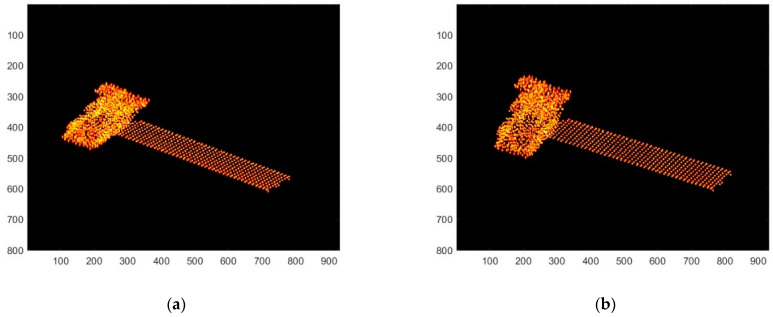
Two-dimensional image. (**a**) First-frame ISAR image; (**b**) Sixth-frame ISAR image.

The 3D structure of the target is shown in Figure 4, and the experimental parameters are shown in Table 1. First, the target is imaged in two dimensions, and the sequence images of six neighboring frames are selected for follow-up study. The ISAR imaging results of the first and sixth frames are shown in Figure 5a,b.

### 5.1. Three-Dimensional Reconstruction of ISAR Sequence Images in High SNR

The image is denoised to separate the object from the background. First, the global threshold is automatically calculated by the Otsu method, and then, the image is binarized to distinguish the target from the background, and finally, the background pixels are removed by mask operation.

#### 5.1.1. ISAR Image Feature Point Extraction and Matching

The feature extraction and association of ISAR sequence images with high SNR are carried out. The Harris algorithm is used for feature point extraction, and the improved KLT algorithm is used for feature point tracking and matching. The number of pyramid layers in the KLT algorithm is three and the number of iterations is ten.

**Figure 6:**
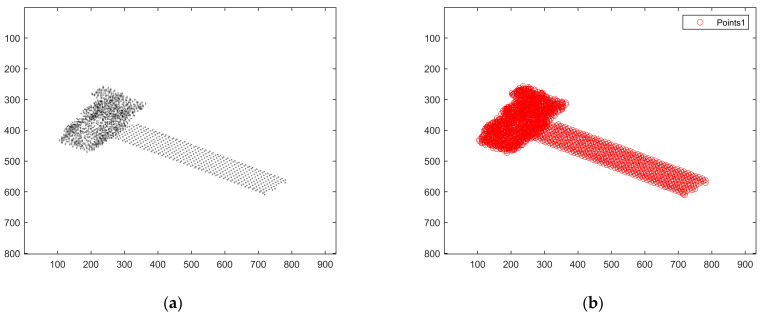
Feature extraction of ISAR images with high SNR. (**a**) The first frame of the ISAR image; (**b**) Feature extraction results of the first frame ISAR image.

**Figure 7:**
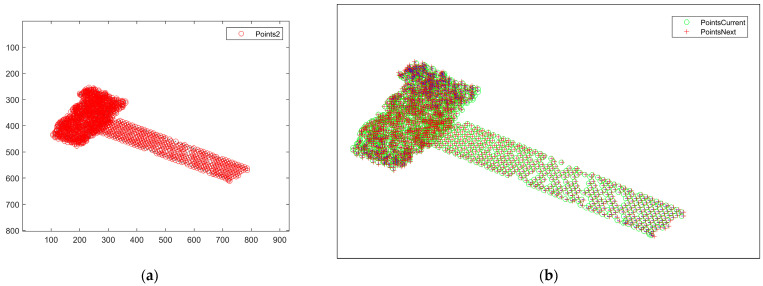
Feature matching and correlation of the first two frames of the image with a high SNR. (**a**) Second-frame ISAR image feature point tracking results. (**b**) Result of feature point association for two neighboring frames.

The result of feature point extraction for the ISAR image is shown in Figure 6, and the association result is shown in Figure 7. Figure 6a is the grayscale image of the first frame of the ISAR image after noise reduction. Feature points are extracted from it, and the result obtained is shown in Figure 6b, where the red circles indicate the extraction results of the feature points in the ISAR image, and it can be seen that the point features of the target in the image can be extracted relatively accurately by the Harris algorithm.

According to the extraction results of the point features, the feature points of the next frame image are tracked, and the simulation image is obtained, as shown in Figure 7a. The location of the red circle indicates the location of the obtained feature point. The result of the feature point association of the two neighboring frames between the first and second frames is plotted in Figure 7b, which can show the motion of feature points between the two frames. In Figure 7b, the green circle marks the feature point in the first-frame image, the red + marks the feature point in the second-frame image, and the matched pairs of points are connected by a short blue line. From the figure, it can be seen that the feature point association result is quite accurate.

#### 5.1.2. Mismatched Point Deletion Based on Motion Vector Features

**Figure 8:**
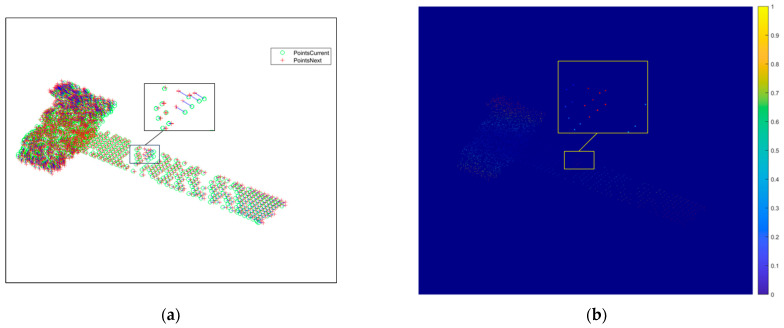
Results before mismatched point deletion between the third- and fourth-frame images. (**a**) Matching point pairs between the third- and fourth-frame images. (**b**) Intensity map.

**Figure 9:**
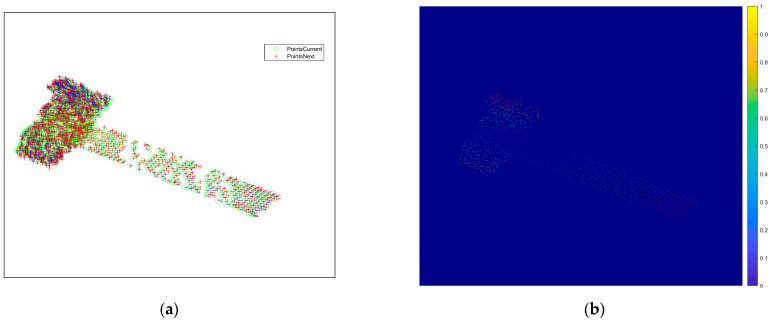
Results after mismatched point deletion between the third- and fourth-frame images. (**a**) Matching point pairs between the third- and fourth-frame images. (**b**) Intensity map.

Figure 8 represents the result of the feature point association between the subsequent third- and fourth-frame images, and it can be seen from Figure 8a that mismatched points exist. Converting the distance and direction to pixel values, the intensity map of the scattering points of the 2D image is plotted as shown in Figure 8b, and there are mismatched points in the zoomed-in area of the yellow boxed line, whose pixel values are different from the surrounding correctly matched points. For the mismatched points that still exist after correlation, the proposed method based on motion vectors is used to delete the mismatched points, and the correlation results after deletion are shown in Figure 9a. It can be seen that there is no region with obvious pixel changes in Figure 9b, which verifies the effectiveness of the proposed method.

**Figure 10:**
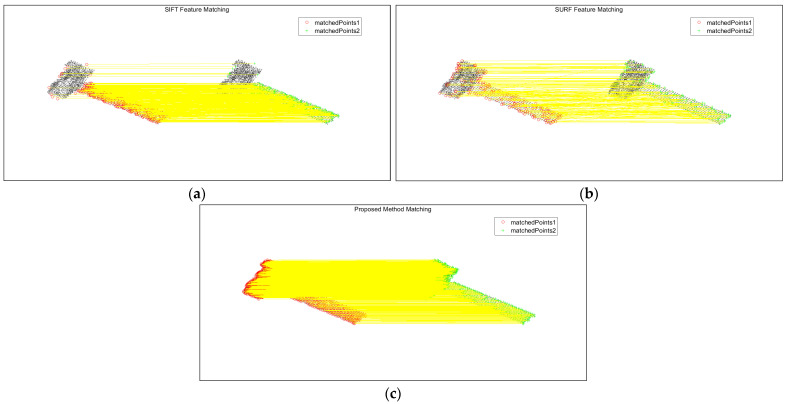
Results of image association between two neighboring frames in high SNR. (**a**) SIFT algorithm; (**b**) SURF algorithm; (**c**) algorithm proposed in this paper.

The method of this paper is compared with the feature point extraction and realization of association matching between two-frame images using the SIFT and SURF methods. Figure 10 shows the association results between the first-frame image and the second-frame image in high SNR, and Figure 10a–c show the SIFT algorithm, the SURF algorithm, and this paper’s algorithm, respectively, and the yellow line connects the corresponding matching feature points in the two-frame images.

As can be seen from the figure, the number of point features extracted by SIFT is not very large, and there are some mismatched points; the number of point features extracted by the SURF algorithm is very small, but the association accuracy in the main part of the body is higher than that of the SIFT algorithm; this paper’s method obtains a significantly larger number of matching point sets than the SIFT algorithm and the SURF algorithm, the matching point pairs are more accurate, and the overall performance is better than that of the first two algorithms. Table 2 records the number of feature points for image association matching by different methods. Only if the number of matching point sets is large enough, the 3D reconstruction can be better, so the algorithm in this paper has a certain superiority in solving the problem of inter-image correlation.

#### 5.1.3. Outlier Point Deletion Based on Local Spatial Point Cloud Density

**Figure 11:**
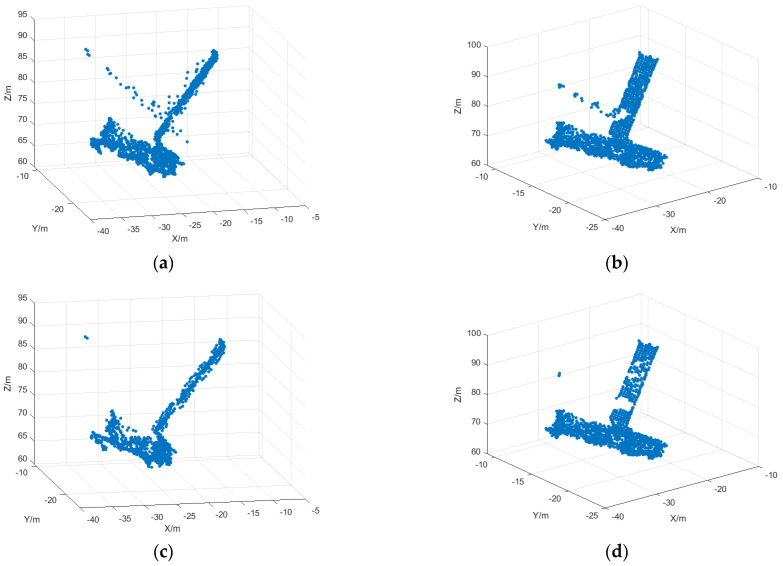

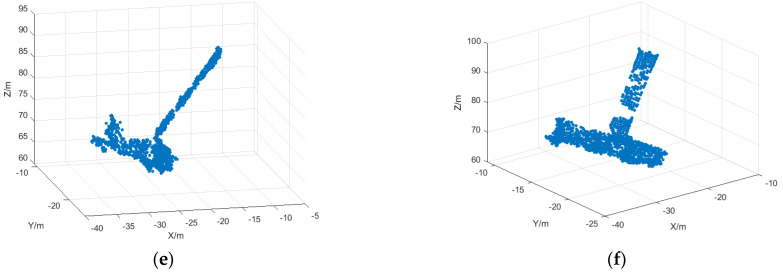
3D reconstruction results based on ISAR sequence images. (**a**) 3D reconstruction after the initial matching of ISAR sequence images, viewpoint 1; (**b**) 3D reconstruction after the initial matching of ISAR sequence images, viewpoint 2; (**c**) 3D reconstruction after the deletion of mismatched points, viewpoint 1; (**d**) 3D reconstruction after the deletion of mismatched points, viewpoint 2; (**e**) 3D reconstruction after outlier deletion, viewpoint 1. (**f**) 3D reconstruction after outlier deletion, viewpoint 2.

The simulation results obtained by 3D reconstruction based on the projection matrix inversion method are shown in Figure 11. Where Figure 11a is the 3D reconstruction result after initial matching, it can be seen that multiple mismatched points exist, and Figure 11b is the 3D reconstruction result after mismatched point deletion based on motion vectors, and it can be seen that due to the reduction in matching mismatched points, the reconstruction result is also more accurate, but error points still exist, so the reconstructed outlying points need to be deleted. Figure 11c shows the reconstruction results after outlier deletion based on the spatial localization point sparsity method, from which it can be found that the outlier points are effectively deleted, and the reconstruction results are more accurate. The number of points in the reconstructed point cloud is shown in Table 3, and it can be observed that the erroneous points have been effectively removed.

### 5.2. Feature Point Matching Based on the KLT Algorithm

**Figure 12:**
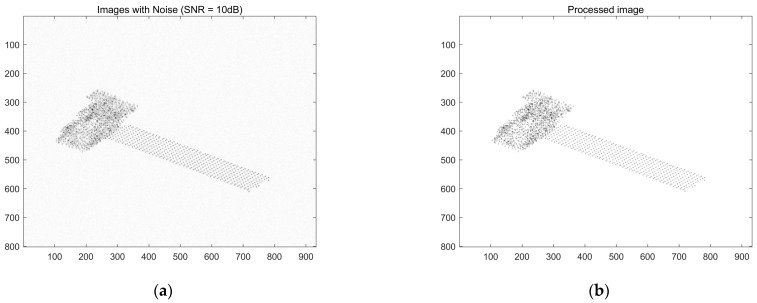
ISAR image processing with SNR = 10 dB. (**a**) First-frame ISAR image; (**b**) First frame after noise reduction.

First, the image is denoised to separate the object from the background. The images of the first ISAR image before and after noise reduction are shown in Figure 12a,b.

#### 5.2.1. ISAR Image Feature Point Extraction and Matching

**Figure 13:**
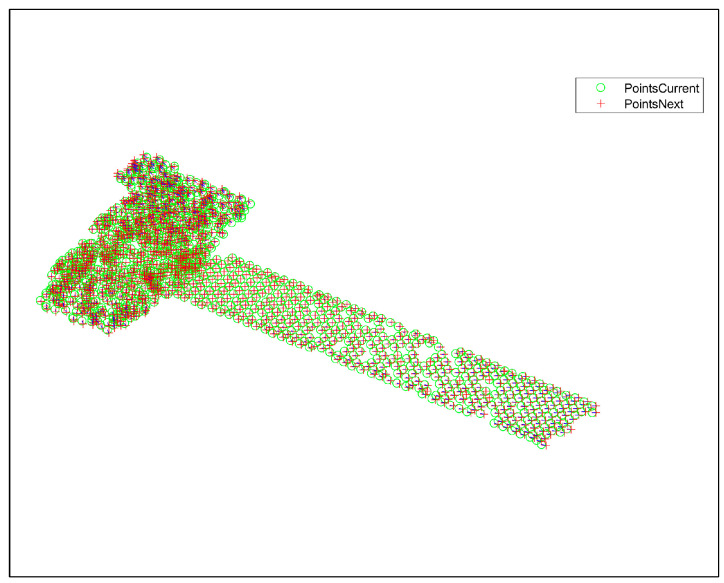
Result of feature point matching for the first two frames of the image with SNR = 10.

The matching results using the first two frames are shown in Figure 13, where the green circle indicates the feature point in the ISAR image of the first frame, and the red + indicates the feature point in the ISAR image of the second frame, and the corresponding matched feature points are connected by a short blue line. It shows from the figure that a sufficient number of feature points can be matched.

#### 5.2.2. Mismatched Point Deletion Based on Motion Vector Features

**Figure 14:**
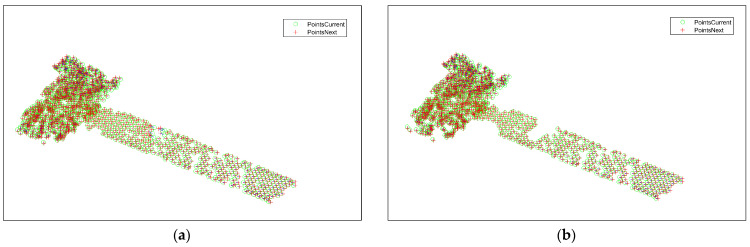
Results before and after mismatched point deletion between the second- and third-frame images. (**a**) Result before mismatched point deletion. (**b**) Result after mismatched point deletion.

Figure 14 shows the result of the feature point association between the subsequent second-frame and the third-frame images, with the presence of mismatched points. For the mismatched points that still exist after association, the proposed motion vector-based method is used to delete the mismatched points. The association result after deletion is shown in Figure 14b, and some mismatched points are deleted, which verifies the effectiveness of the proposed method.

Table 4 shows the number of association matching points for the first two frames of the SIFT algorithm, the SURF algorithm, and the algorithm of this paper at different SNRs. It shows that the algorithm in this paper obtains a higher number of matching points than the other two algorithms.

#### 5.2.3. Outlier Point Deletion Based on Local Spatial Point Cloud Density

**Figure 15:**
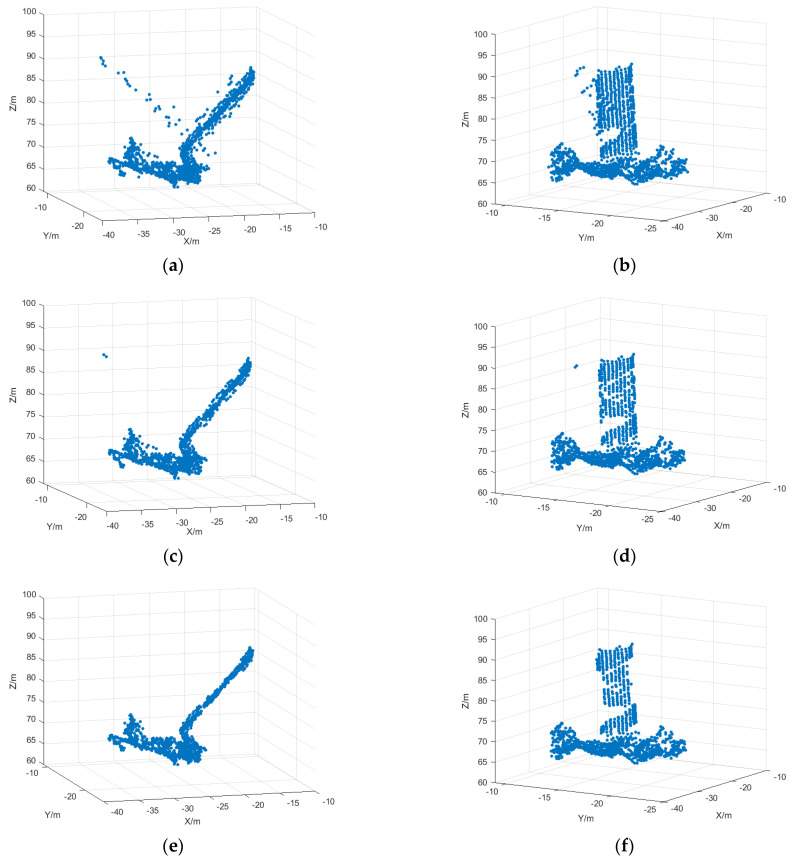
3D reconstruction results based on ISAR sequence images. (**a**) 3D reconstruction after initial matching of ISAR sequence images, viewpoint 1; (**b**) 3D reconstruction after initial matching of ISAR sequence images, viewpoint 2; (**c**) 3D reconstruction after the deletion of mismatched points, viewpoint 1; (**d**) 3D reconstruction after the deletion of mismatched points, viewpoint 2; (**e**) 3D reconstruction after outlier deletion, viewpoint 1; (**f**) 3D reconstruction after outlier deletion, viewpoint 2.

The simulation results obtained by the three-dimensional reconstruction of the ISAR image sequence with SNR = 5 are shown in Figure 15. The number of points in the reconstructed point cloud is shown in Table 5, where Figure 15a,b is the 3D reconstruction result after the initial matching based on the initial matching, and Figure 15c,d is the 3D reconstruction result after the deletion of the mismatched points based on the motion vectors. It can be seen that the reconstruction results are also more accurate due to the reduction in matching mismatched points, but there are still mismatched points present. Figure 15e,f shows the reconstruction result after outlier deletion after reconstruction, and it can be seen from the figure that the outlier points are effectively deleted and the reconstruction result is more accurate.

However, the effect of reconstruction is not as good as that in the case of a high SNR. The reason may be that in a low SNR environment, noise will drown the weak gradient region in the image, which may interfere with the detection of feature points, and the tracking error of the KLT algorithm may increase, resulting in a reduction in tracked points.

## 6. Conclusions

In this paper, the problem of error point deletion in the 3D image reconstruction of the ISAR sequence is studied, and the methods of mismatched point and outlier point deletion are proposed. First, for the feature point extraction and matching problem, it is solved by the Harris corner point detection algorithm and the improved KLT algorithm. Then, for the possible mismatched points after association matching, a deletion strategy based on motion vector features is proposed, which deletes the mismatched point pairs and improves the matching accuracy by comprehensively considering the double constraints of motion distance and direction. After the 3D reconstruction is completed, the existing mismatched points need to be further processed. Thus, a threshold judgment method based on the local spatial point density is introduced, which is able to identify and delete outlier points to improve the accuracy of the target 3D reconstruction results. Finally, simulation experiments are carried out on the satellite model to reconstruct the ISAR 2D sequence images in 3D under the condition of a high SNR and an SNR = 10, respectively. The correctness and effectiveness of the proposed method are verified by the simulation experiment. This paper analyzes and processes the simulation model under ideal conditions. However, in practical observations, components may be occluded. Effectively recovering the information of the occluded components is a problem that requires in-depth consideration and research in future work. Additionally, future work could involve exploring the optimal density of the point cloud.

## Figures and Tables

**Table 1:** ISAR imaging parameters.

Parameters	Values
PRF	75 Hz
Pulse width	25 µs
Bandwidth	3 GHz
Center frequency	35 GHz

**Table 2:** The number of image-association-matching feature points by different methods.

Method	Feature Points Number
SIFT	664
SURF	178
Proposed Method	1597

**Table 3:** The number of points in the 3D reconstructed point cloud.

Method	Number of Points
Initial 3D reconstruction	1644
After mismatched point deletion	1483
After outlier deletion	1432

**Table 4:** The number of image-association-matching feature points by different methods.

Method	Feature Points Number	
	SNR = 10	SNR = 5
SIFT	235	216
SURF	29	38
Proposed Method	1256	978

**Table 5:** The number of points in the 3D reconstructed point cloud.

Method	Number of Points
Initial 3D reconstruction	1323
After mismatched point deletion	1165
After outlier deletion	1108

## Data Availability

Data are available on request from the authors.
